# Acquisition of durable insulin-producing cells from human adipose tissue-derived mesenchymal stem cells as a foundation for cell- based therapy of diabetes mellitus

**DOI:** 10.1038/s41598-024-74527-w

**Published:** 2024-10-18

**Authors:** Ghada Nour Eldeen, Hadeer A. Aglan, Nadia S. Mahmoud, Mazen Abdel Rasheed, Osama M. Azmy, Hanaa H. Ahmed

**Affiliations:** 1 Stem Cell Research Group, Medical Research Center of Excellence, National Research Centre, Dokki, Giza Egypt; 2 Department of Molecular Genetics and Enzymology, Human Genetic and Genome Research Institute, National Research Centre, Dokki, Giza Egypt; 3 Hormones Department, Medical Research and Clinical Studies Institute, National Research Centre, 33 El Buhouth St. (Former El- Tahrir St.), P.O. 12622, Dokki, Giza Egypt; 4 Stem Cell Lab, Center of Excellence for Advanced Sciences, National Research Centre, Dokki, Giza Egypt; 5 Department of Reproductive Health Research, Medical Research and Clinical Studies Institute, National Research Centre, Dokki, Giza Egypt; 6 Egypt Center for Medical Research and Regenerative Medicine, El Shorouk, Egypt

**Keywords:** Human adipose tissue derived stem cells, Laminin–coated plates with extrinsic factors, Insulin- producing cells, *In vitro* assessment, *In vivo* study, Biochemistry, Stem cells

## Abstract

This study aimed to identify the suitable induction protocol to produce highly qualified insulin producing cells (IPCs) from human adipose tissue derived stem cells (ADSCs) and evaluate the efficacy of the most functionally IPCs in management of diabetes mellitus (DM) in rats. The ADSCs were isolated and characterized according to the standard guidelines. ADSCs were further induced to be IPCs in vitro using three different protocols. The success of trans-differentiation was assessed in vitro through analysis of pancreatic endocrine genes expression, and insulin release in response to glucose stimulation. Then, the functionalization of the generated IPCs was evaluated *in vivo.* The in vitro findings revealed that the laminin-coated plates in combination with insulin-transferrin-selenium, B27, N2, and nicotinamide could efficiently up-regulate the expression of pancreatic endocrine genes. The in vivo study indicated effectual homing of the PKH-26-labelled IPCs in the pancreas of treated animals. Moreover, IPCs infusion in diabetic rats induced significant improvement in the metabolic parameters and prompted considerable up-regulation in the expression of the pancreatic related genes. The regenerative effect of infused IPCs was determined through histological examination of pancreatic tissue. Conclusively, the utilization of laminin–coated plates in concomitant with extrinsic factors promoting proliferation and differentiation of ADSCs could efficiently generate functional IPCs.

## Introduction

Diabetes mellitus (DM) is a lifelong systemic disorder caused by impaired insulin metabolic mode of action resulting in impaired glucose homeostasis. Long term uncontrolled diabetes with high blood glucose level causing what so called metainflammation^1^, with consequences of serious complications and multisystem deterioration. Management of DM mainly includes lifestyle interception, insulin replacement therapy, and imitative oral hypoglycemic agents^2^. Islet cell transplantation seemed to be the definitive approach for treatment but scarcity of donors prohibited it^1^.

Mesenchymal stem cells (MSCs)-derived β cells are currently the most acceptable alternate as a source of cells. Mesenchymal stem cells are uncommitted multipotent cells resident in body tissues helping in repair mechanisms. According to the modern medical science, these cells have great potential as cell-based therapies. According to the Internal Fat Applied Technology Society, adipose tissue-derived stem cells (ADSCs) exhibited a potential of differentiation similar to MSCs and express the specific stem cell markers in vivo^3^. Interestingly, adipose tissue derived stromal cells are considered as substantial clinical cell source and introduce a clear priority over bone marrow-derived MSCs^4,5^. ADSCs are isolated from subcutaneous fat with minimal invasive procedures and are known to be an affluent exporter of stromal cells regarding harvest and cell yield. Over the past few years, remarkable interests in ADSCs-based therapies have developed. The therapeutic actions of these cells were illustrated for several diseases including wound healing^6^, cardiovascular disease^7^ and cartilage regeneration^8^. In vitro differentiation potential of MSCs depends on adjusted modulations of culture media or even by direct genetic manipulations^9^.

Adequate trans-differentiation of stem cells towards IPCs could be the hallmark in recuperation of diabetic state. Competence of stem cell differentiation is regulated by both internal genetic programming and external stimuli from the microenvironment. Several biological cascades triggered by extracellular matrix (ECM) proteins, cell-to-cell contact, and soluble factors, promoting stem cell differentiation potential^10^. Standard protocols are now available for MSCs differentiation to certain cell lineage such as osteogenic, chondrogenic, and adipogenic cell lines. Several studies have reported protocols for differentiation of MSCs into IPCs. The differentiation protocols of MSCs into insulin producing cells (IPCs) show a great diversity^11–13^. This study aims to identify which is the optimum protocol to generate highly qualified IPCs derived from MSCs of human adipose tissue origin. To achieve this aim, three distinct protocols were applied and assessed using in vitro parameters particularly insulin related genes and insulin release assay to nominate the most promising protocol to be applied in in vivo study to evaluate the efficacy of IPCs generated from the durable protocol as cell-based therapy for DM using STZ diabetic rat model.

## Materials and methods

### Isolation and expansion of human adipose-derived stem cells (ADSCs)

The research study was approved by the Ethics Committee for Medical Research of National Research Centre (Approval Number 19/109). Subcutaneous fat was obtained from twelve female donors aged between 22 and 35 years’ undergoing caesarian sections. Informed consent was obtained from all participants. Cells were dissociated from fat tissue using type I collagenase (Gibco, Cat # 17018029, USA). The isolated stromal vascular fraction (SVF) was cultured in complete medium of DMEM: F12 1:1 (Gibco, Cat # 12500062) medium supplemented with 10% fetal bovine serum (FBS), (Gibco, Cat # 12662029), 2 mM L-glutamine, and Antibiotic-Antimycotic (100X) (Gibco, Cat # 15240096), and incubated at 37 °C and 5% CO_2_ humidified atmosphere, to obtain adherent cells. Non-adherent cells were washed after 72 h and adherent cells were remained in complete medium for 2 weeks. The culture medium was refreshed twice a week. After reaching nearly 70–80% confluent cells, the cells were detached using 0.25% trypsin\EDTA for passaging. Cells were observed by inverted microscope examination^14^.

### Flow cytometric analysis

Isolated ADSCs at passage 3 were trypsinzed for immune-phenotypic analysis. The cells were incubated with fluorochrome-conjugated monoclonal antibodies, according to the manufacturer’s instructions, at 4 °C for 30 min in the dark. The antibodies used were anti-human, CD90-PE, CD73-PE, CD34-PE, CD14-PE, CD105-FITC, and CD45-FITC (BD Biosciences, USA). The labeled cells were analyzed on a FACS Caliber (Becton-Dickinson, FAC scan, San Jose, CA, USA)^15^.

### Multi-lineage differentiation potential

The isolated ADSCs were induced to differentiate into adipocytes, chondrocytes, and osteocytes, using StemPro^®^ adipogenesis differentiation medium, StemPro^®^ chondrogenesis differentiation medium, and StemPro^®^ osteogenesis differentiation medium (Gibco, Thermo Fisher Scientific Inc. (USA), respectively. For each protocol, differentiation medium was placed in experimental wells and standard ADSC medium in control wells. Induction was performed according to the manufacturer’s instructions. Oil-Red-O stain (Sigma-Aldrich, St. Louis, MO, USA) was used to determine adipocytes phenotype, Alcian blue stain (Sigma-Aldrich) for chondrocytes, and Alizarin-red (Sigma-Aldrich) staining was used to detect calcium deposits of osteocytes^16^.

### Differentiation of ADSCs into IPCs

IPCs-differentiation induction was established by three different protocols.

*The first protocol (P1)* was implemented according to^17^. As MSCs were primarily potentiated using 10 mmol/L nicotinamide (Sigma-Aldrich) and 1 mmol/L β-mercaptoethanol (Sigma-Aldrich) in 10% FBS LG-DMEM (Serox, Germany) for the first 48 h. The medium was replaced by 10 mmol/L nicotinamide and 1 mmol/L β-mercaptoethanol in serum-free high glucose (HG)-DMEM (Serox) for another 24 h. Further induction for the next 7 days were performed by 10 nmol/L exendin-4 (Sigma-Aldrich) in serum free HG-DMEM supplemented with 10 mmol/L nicotinamide and 1 mmol/L β-mercaptoethanol.

*The second protocol (P2)* was described by^18^ and started with passaging of MSCs on Geltrex™ (Gibco)-coated 6-well culture plates in LG-DMEM containing 15% FBS for 24 h. The medium was replaced by HG-DMEM, containing 2% FBS and 100 ng/ml activin A (STEMCELL Technologies Inc, Canada) for the next 24 h. and then changed to HG-DMEM containing 2% FBS and 10^− 6^ mol/l retinoic acid (RA; Sigma Aldrich) for an additional 24 h. Subsequently, the cells were cultured in HG-DMEM containing 2% FBS and 10 ng/ml basic fibroblast growth factor (bFGF; STEMCELL Technologies Inc) for another 3 days. The culture medium was replaced by HG-DMEM containing 2% FBS and 10 mmol/l nicotinamide for 3 days. Eventually, the cells were cultured in HG-DMEM containing 2% FBS and 10 mmol/l nicotinamide in the presence of 10 mmol/l exendin-4 for 3 days.

*The third protocol (P3)* was carried out according to study design previously reported by^19^; aliquots of 2.5 × 10^5^ cell suspension in complete culture media were plated in 5 µg/mL laminin (Gibco) pre-coated 6-well plates overnight. At stage I, the medium was replaced by fresh HG-DMEM containing 10% FBS for 3 d. The media were refreshed with DMEM/F-12 (Lonza) medium containing 2% FBS and 1% Insulin-Transferrin-Selenium-A (ITS-A, Gibco) for another 4 d. At stage III, 10 mM nicotinamide was added into the media described in stage II, and the culture lasted for 3 days. Eventually, at stage IV, the medium was removed and refreshed with a new medium containing the same supplements as at stage III, and 1% N2 and 1% B27 supplements (STEMCELL Technologies Inc) were included and incubated for 4 d.

### Quantitative analysis of β cell-related genes

Total RNA was purified from induced IPCs differentiated by the three different protocols using TRI reagent and Direct-Zol RNA kit (ZYMO RESEARCH, USA) according to the manufacturer’s instruction. Furthermore, cDNA was synthesized (COSMO cDNA synthesis kit, Willowfort, UK) using 100 ng of purified RNA as a template and random hexamers with oligo (dT) primers to initiate reverse transcription reaction. Gene expression was performed for endocrine hormones markers (*insulin (INS)*, and *glucagon (Gcg)*), and, pancreatic–specified transcription factors (*pancreatic and duodenal homeobox 1* (*PDX-1*), *Neurogenin 3* (*Ngn 3*), *forkhead box protein A2 (Foxa-2*), and *SRY-box transcription factor 17* (*Sox-17*)). SYBER Green-based qPCR reactions were implemented in a total volume of 20 µL containing 10 µM of each primer, 10µL of 2xSensiFAST SYBER Lo-ROX and 1µL of cDNA. PCR reactions were provided in duplicate and heated to 95°C for 10 min followed by 40 cycles of denaturation at 95°C for 15 seconds, annealing at 58–60°C for 1 min, and extension at 72°C for 20 seconds. 2^−ΔΔCt^ comparative cycle time (CT) was anticipated to define fold differences between samples and normalized to an endogenous reference (*GAPDH*). The primer sequence for human marker genes were as follows: *Foxa-2*, F;5’- GGAGCGGTGAAGATGGAAGG-3’, and R; 5’-CGGCGTTCATGTTGCTCAC-3’, *sox-17*, F; 5’-CAAGATGCTGGGCAAGTC-3’, and R; 5’-TGGTCCTGCATGTGCTG-3’, *PDX-1*, F; 5’-ATGGATGAAGTCTACCAAAGC-3’, and R; 5’- CGTGAGATGTACTTGTTGAATAG-3’^20^, *Ngn3*, F;5’-CGCCGGTAGAAAGGATGAC-3’ and R; 5’-GAGTTGAGGTTGTGCATTCG-3’^21^; *Glucagon*, F; 5’-ACCAGAAGACAGCAGAAATG − 3’, and R; 5’-GAATGTGCCCTGTGAATG − 3’, *Insulin*, 5’-AACCAACACCTGTGCGGCTCA-3’ and 5’-TGCCTGCGGGCTGCGTCTA-3’^20^; and *GAPDH*, F; 5’-GCACCGTCAAGGCTAGAAC-3’, and R; 5’-TGGTGAAGACGCCAGTGGA-3’^22^.

### In vitro glucose-stimulated insulin secretion (GSIS) assay

Different generated IPCs were gently washed twice with phosphate buffer saline (PBS, Biowest, France). Then, the cells were pre-incubated in culture media containing glucose (5.5 mM) at 37 °C for 24 h^23^ and supernatants were then collected for insulin quantification *via* ELISA (Epitope Diagnostics, Inc., USA) according to the manufacturer’s instructions. Insulin levels were calculated according to the standard curve.

### Labeling of the generated IPCs with PKH26 dye

The most functionally generated IPCs were harvested and labeled with PKH26 (Sigma-Aldrich, USA) fluorescent linker dye according to the manufacturer’s instructions. STZ- induced diabetic rats were infused with IPCs- labelled cells (5 × 10^6^ cells/rat). Later, experimental animals were sacrificed and pancreatic tissues were examined using fluorescence microscope (Olympus, CKX41, Japan) to detect and trace the cells.

### Induction of diabetes mellitus and treatment of diabetic rats with IPCs

#### Animals

Adult male albino *Wistar* rats (*n* = 30) weighing 150–170 g were obtained from the Animal Care Unit of the National Research Centre, Giza, Egypt. Rats were maintained under standard complete sterile conditions in a temperature-stabilized room with a 12 h light/dark schedule, and were allowed for free nourishment. All experimental animals has been given 1-week acclimatization period before experiment. The experimental procedures were established in compliance with the Institutional Ethical Committee guidelines.

#### IPCs infusion

At the end of the acclimatization period, the animals were classified into 3 groups. Group 1 (*n* = 10), served as negative control group and received no drugs. Group 2&3 were induced to be diabetic models through single infusion of 50 mg/kg streptozotocin (STZ, Sigma, USA) following an overnight fast. Fasting blood glucose (FBG) was measured after 72 h from injection. It was considered as a state of diabetes when the blood glucose level reached 250 mg/dl. Group 2 (*n* = 10), was left untreated (DM untreated), while in Group 3 (*n* = 10), 5 × 10^6^ cells/rat of IPCs produced by selected protocol were infused *via* tail veins (DM + IPCs). Four weeks after infusion, IPCs therapeutic efficacy was evaluated^24^.

### Functional analysis of infused IPCs

According to the manufacturer’s guidelines, blood glucose level (Chemelex S.A., Spain) was estimated. Serum insulin (INS), c-peptide (CP) and visfatin levels as well as pancreatic glucagon (GC) level were also quantified by ELISA kits (SinGeneClon Biotech Co., Ltd, China).

### Molecular genetic analysis

Total RNA was isolated from pancreatic tissue of rats of different experimental groups using trizol reagent (Invitrogen, USA.) in combination with RNeasy mini kit for total RNA purification from animal cells (Qiagen, Germany) according to the manufacturer’s instruction. RNA integrity was evaluated using Nano Drop 2000 (Thermo Fisher Scientific, Rockford, IL, USA) using 260/280 nm ratio. Then, cDNA synthesis was performed using Revert Aid first strand cDNA synthesis kit (Thermo Fisher Scientific, Inc., Lithuania) according to the manufacturer’s instruction. Gene expression analysis of *Foxa2* and *Sox17*, *insulin-like growth factor I* (*IGF-1*) and *fibroblast growth factor* (*FGF*) *10* was carried out in rat pancreatic tissues using DNA-Technology Real-Time PCR device (DT lite 4, Russia). The reaction mixture (25 µl volume) included 12.5 µl of QuantiTect SYBR Green master mix (Qiagen), 0.75 µl of forward and reverse primer of target gene (Invitrogen), cDNA template (100 ng) and RNase free water. GAPDH was used as a housekeeping gene. The primer sequence for rat gene markers were as follows: *Foxa-2*, F; 5’-TGAAGCCCGAGCACCATTAC-3’, and R; 5’-CCAGGGTAGTGCATGACCTGTT-3’^25^; *Sox-17*, F; 5’- GGCGCCAGCCGGGACCTC-3’, and R; 5’- GGCCGCCCTCGGGACCAA − 3’^18^, *IGF-1;* F; 5’-GCTTTTACTTTCAACAAGCCCACA-3’, and R; 5’- TCAGCGGAGCACAGTACATC − 3’, *FGF-10;* F; 5’- TTGCTCTTCTTGGTGTCTTCC − 3’, and R; 5’- ACCTTGCCGTTCTTTTCAATC − 3’^26^, *GAPDH*, F; 5’- CACCCTGTTGCTAGCCATATTC-3’, and R; 5’- GACATCAAGAAGGTGGTGAAGCAG − 3’^27^. Relative mRNA expression *versus* control value was assessed using the 2^-ΔΔCt^ comparative method after normalization with GAPDH gene. The PCR cycling was set as follows: initial denaturation step at 94ºC for 15 min, followed by 40 cycles of denaturation at 94 °C for 15 s, annealing at 60 °C for 30 s and extension at 72 °C for 30 s for 5 min. Data were presented as the fold change in gene expression level in the diabetic group relative to the control group. While, data were represented as the fold change in gene expression as compared to diabetic group in case of treated group.

### Histological inquiry of pancreas tissue

Pancreas tissues were fixed in 10% formalin overnight, and then embedded in paraffin wax. 5–6 microns’ thickness- tissue blocks were provided to be stained with Hematoxylin and Eosin stain (H&E) for further histological examination according to standard protocols^28^. The morphological structure of each tissue sample was demonstrated under a light microscope (Olympus, Japan).

### Statistics

Statistical parameter’s estimation was provided as arithmetic means with their standard deviations (S.D). Statistical significance among groups was analyzed by one-way analysis of variance (ANOVA) test using the Statistical Package for the Social Sciences (SPSS) 20 followed by defining the least significant difference (LSD). A value of *P* < 0.05 was rated to elucidate statistical significance.

## Results

### ADSCs phenotypic aspects

**Fig. 1:**
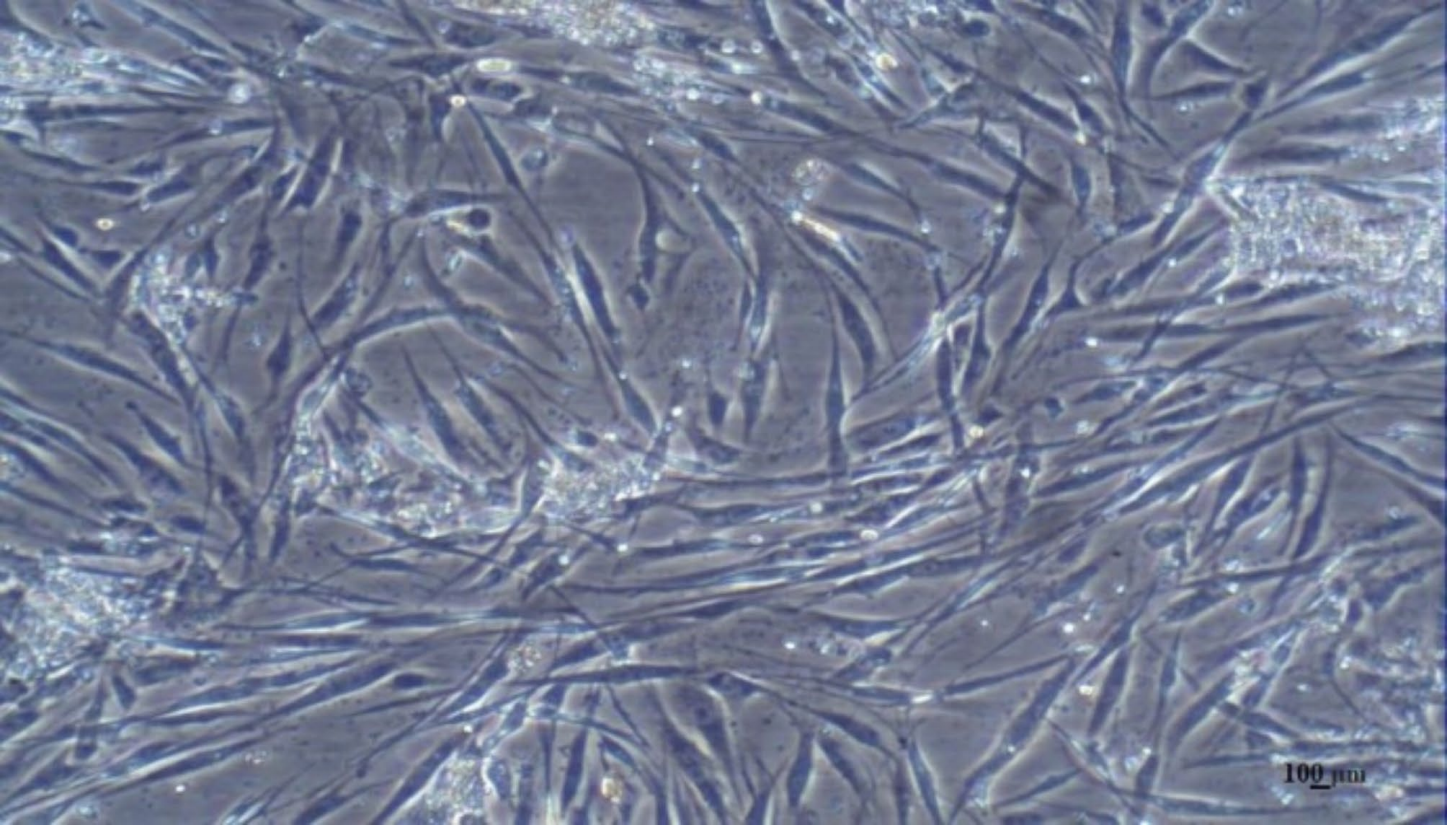
Morphological aspects of isolated MSCs from human adipose tissue at passage 3.

**Fig. 2:**
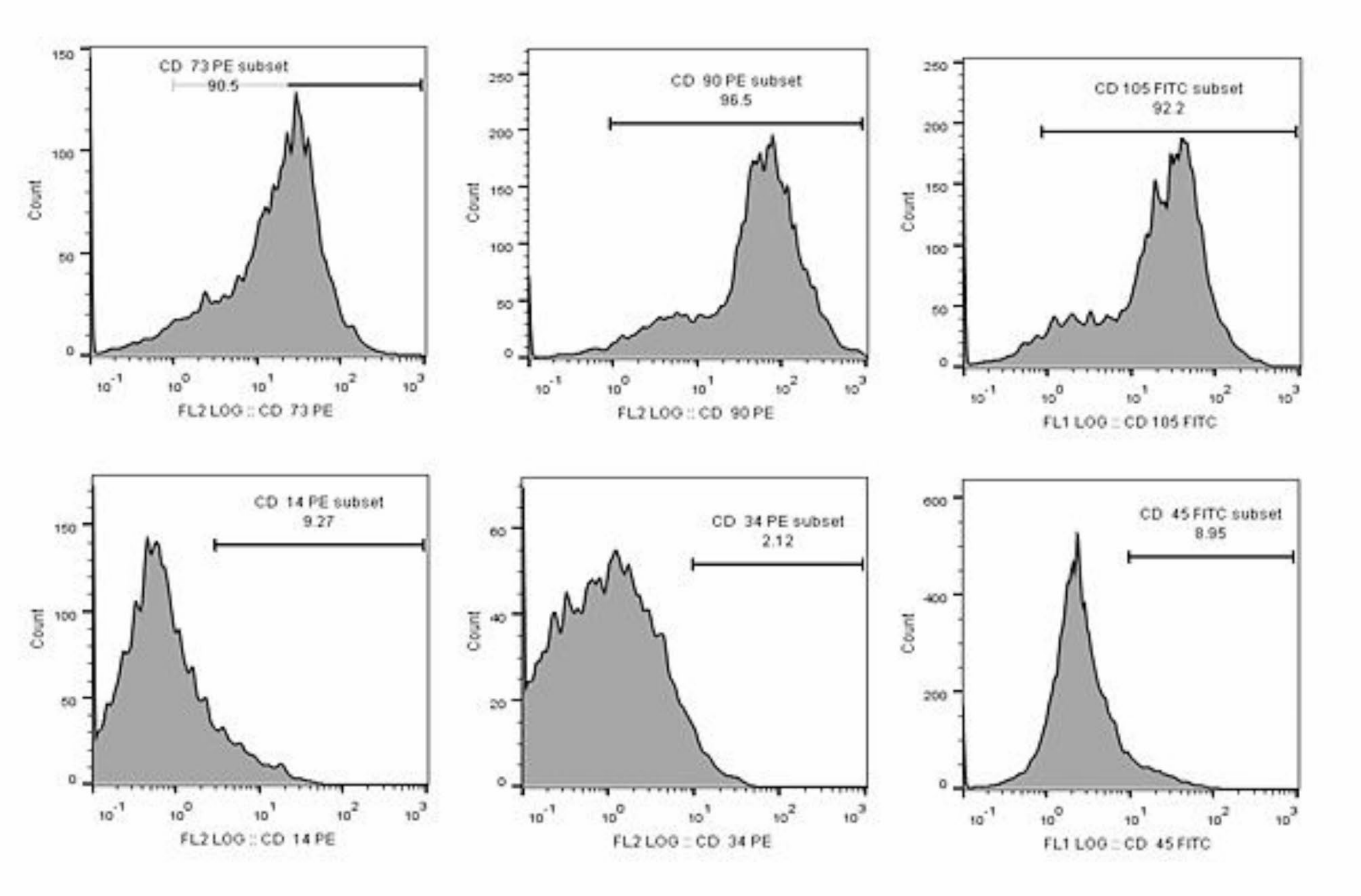
Flow cytometric analysis of human adipose tissue derived MSCs after staining with CD 73, 90, 105, 14, 34 and 45.

Morphologically, adherent fusiform-like cells were appeared as early as 5–7 days after cultivation. As displayed in Fig. 1, the photographed cells were almost homogenous with fibroblast-like performance. Furthermore, immune-phenotypic analysis revealed that the cultured cells were positive for mesenchymal stem cell markers, CD 73 (90.5%), CD 90 (96.5%) and CD 105 (92.2%) but they were negative for hematopoietic stem cell surface markers, CD 14 (9.27%), CD 34 (2.12%) and CD 45 (8.95%) (Fig. 2).

### Tri-lineage mesodermal differentiation

**Fig. 3:**
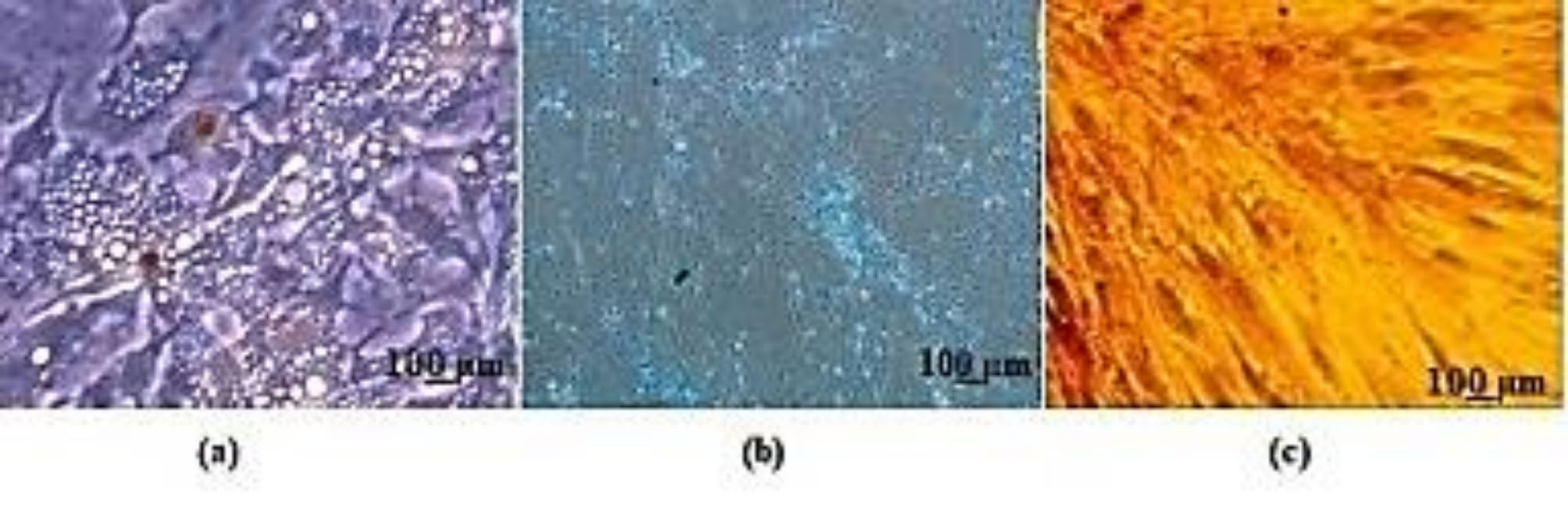
Photomicrographs of MSCs derived from human adipose tissue differentiation into (**a**) adipocytes, (**b**) chondrocytes and (**c**) osteoblasts.

The functional assessment of adipose tissue derived cells was determined by evaluating the mesodermal differentiation prospect of these cells. As presented in Fig. 3a-c, these ADSCs showed adipocyte-like cell obtainment; confirmed by Oil Red staining of lipid droplets. Furthermore, these cells displayed an osteocyte-like cells; demonstrated by Alizarin Red-S staining for calcium-rich extracellular matrix. Eventually, the chondrogenic differentiation potential of the cultured ADSCs was emphasized by Alcian blue staining for sulfated proteoglycan.

### Durability of induced IPCs

#### Transcriptional expression of insulin-related genes

**Fig. 4:**
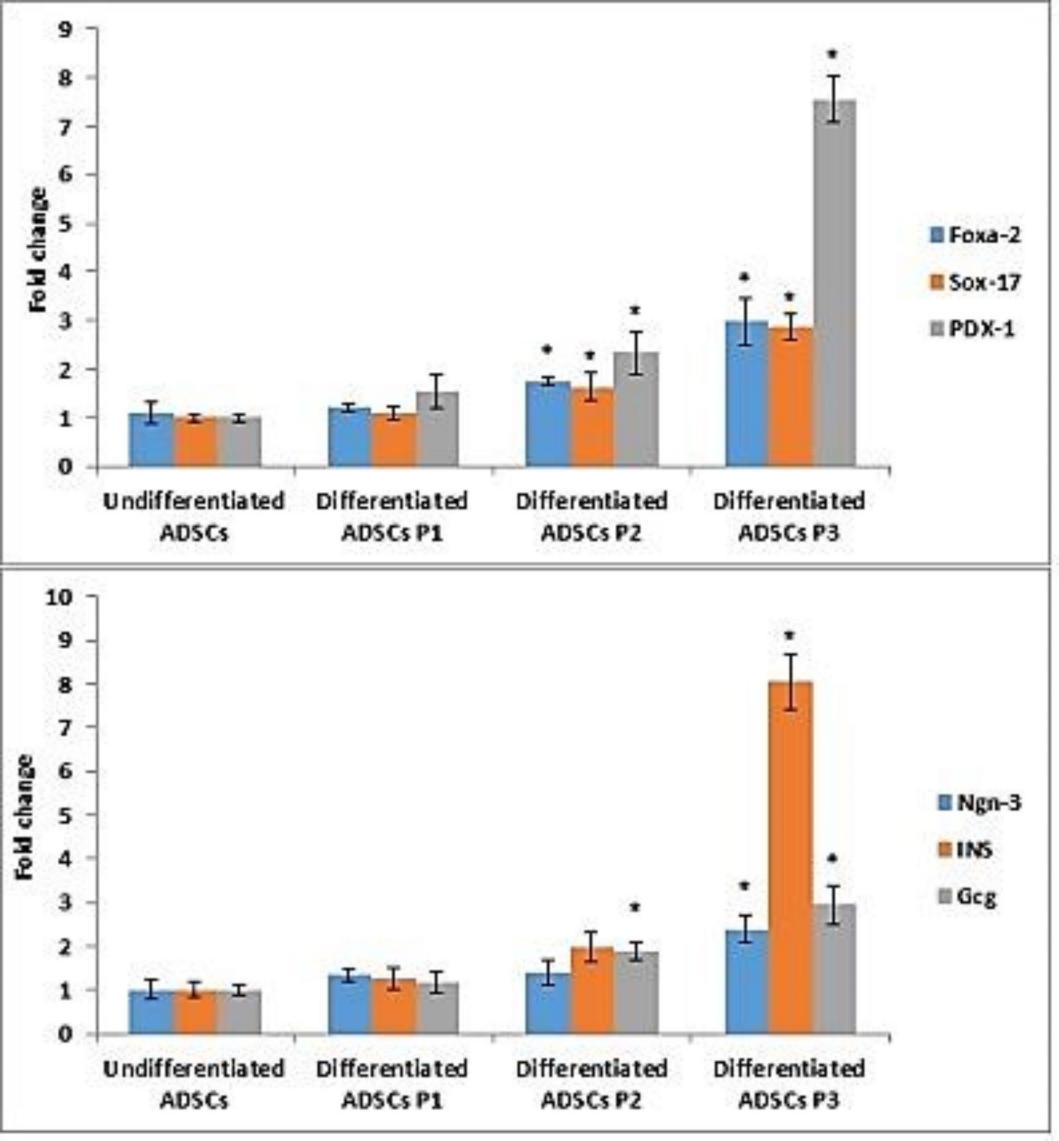
Genetic markers expression of acquired IPCs from ADSCs by the suggested protocols. Data are expressed as Means ± SD. *Significant change at *P* < 0.05 in comparison with the undifferentiated ADSCs.

The mRNA levels of expression of β-cell-related genes were quite diverse between the three induction protocols. As illustrated in Fig. 4, the induced cells derived from protocol 1 showed insignificant (*P* > 0.05) up-regulation in the expression level of *Foxa-2*,* Sox-17*,* PDX-1*,* Ngn-3*,* insulin* and *glucagon* genes relative to undifferentiated ADSCs. Moreover, ADSCs exposed to protocol 2 expressed significant (*P* < 0.05) up-regulation of *Foxa-2*,* Sox-17*,* PDX-1* and *glucagon* genes and insignificant (*P* > 0.05) up-regulation in the expression level of *Ngn-3* and *insulin* genes in comparison with undifferentiated cells. Finally, the generated IPCs upon culturing MSCs derived from human adipose tissue with the ingredients of protocol 3 displayed profound significant (*P* < 0.05) up-regulation in the expression level of pancreatic endocrine genes (*Foxa-2*,* Sox-17*,* PDX-1*,* Ngn-3*,* insulin* and *glucagon*) *versus* undifferentiated ADSCs.

#### Functional feasibility of induced IPCs

**Fig. 5:**
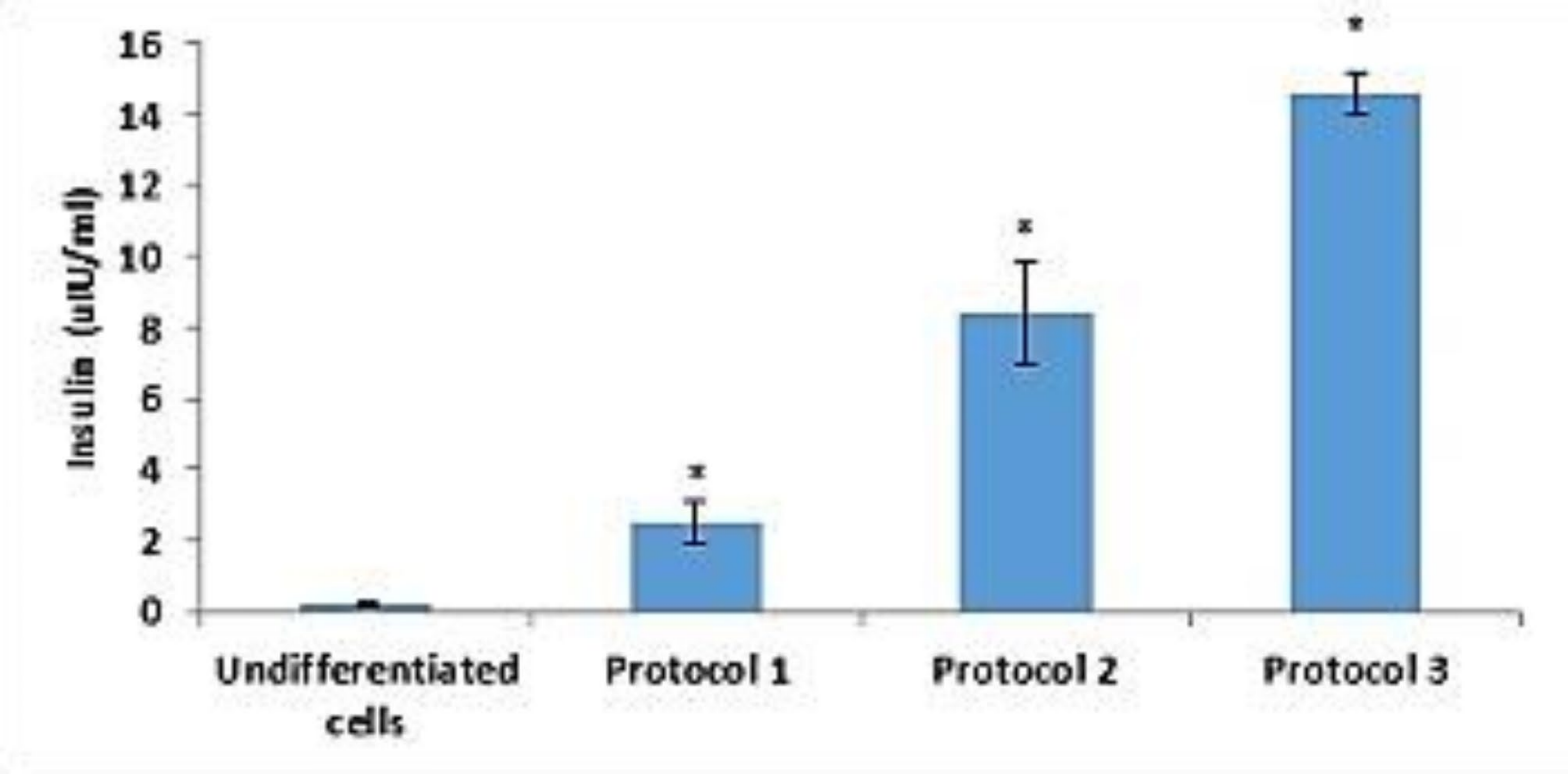
Insulin release of generated IPCs from ADSCs by different protocols. Data are expressed as Means ± SD. *Significant change at *P* < 0.05 in comparison with the undifferentiated ADSCs.

Functional assessment of the IPCs generated with these protocols, was indicated by glucose-stimulated insulin secretion (GSIS) for the developed IPCs. As shown in Fig. 5, the obtained IPCs from the selected protocols released significant (*P* < 0.05) amounts of insulin in response to increasing glucose concentration *versus* their undifferentiated counterparts. Moreover, insulin secreted by the protocol 3–induced cells was remarkably higher than that produced by cells initiated by other protocols in response to increasing glucose concentration.

### Tracking of transfused IPCs

**Fig. 6:**
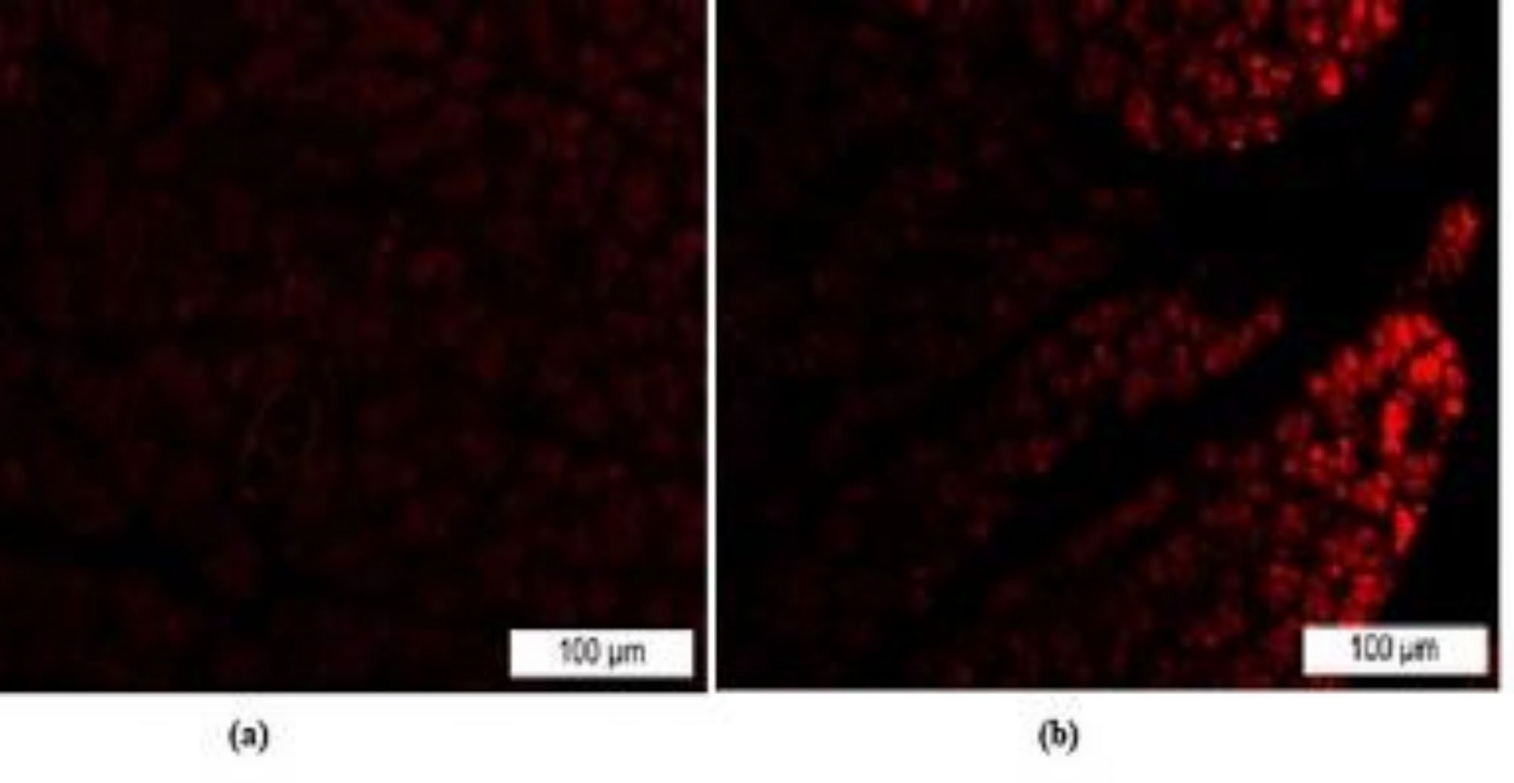
Homing of transfused IPCs in pancreas tissue of treated group. (**a**) Fluorescent micrograph of pancreas tissue from DM untreated rat. (**b**) Fluorescent micrograph of pancreas tissue from DM rat transplanted with IPCs from ADSCs differentiation.

The homing of transplanted IPCs was assessed in pancreas tissue after injection of PKH-26 labeled IPCs. Fluorescent photographs showed that there were many PKH-26-labeled cells in pancreas sections of treated group with the generated IPCs from ADSCs (Fig. 6).

### Assessment of in vivo experimental studies

#### Diabetic metabolic parameters

Functional evaluation of the infused –IPCs was estimated through measurement of blood glucose level, serum insulin (INS), c-peptide (CP) and visfatin (VF) as well as pancreatic glucagon (GC) levels. Metabolic parameters were monitored of negative control group to be calculated as standard estimated parameters. Table 1 illustrated the value of measured parameters for untreated STZ-induced diabetic rats and that of diabetic rats received *in vitro-* induced IPCs.

**Table 1:** Effect of IPCs transplantation on diabetic parameters, blood glucose level, serum INS, CP, VF, and pancreatic GC levels. Data are expressed as means ± SD of 10 rats/group.

Groups	Parameters				
	Glucose (mg/dL)	INS (mU/L)	CP (pg/mL)	Visfatin (ng/mL)	GC (pg/ g tissue)
Negative control	96.5 ± 9.45	13.5 ± 2.86	897.5 ± 67.1	156.3 ± 10.6	161.3 ± 21.6
DM untreated	310.9 ± 53.6^a^	4.1 ± 0.97^a^	370.6 ± 71.8^a^	244.9 ± 24.2^a^	320.8 ± 22.5^a^
DM + IPCs	112.4 ± 21.3^b^	10.1 ± 2.2^b^	728.1 ± 194.9^b^	165.4 ± 12.7^b^	202.5 ± 28.4^b^

^a^Significant change at *P* < 0.05 in comparison with the negative control group.

^b^Significant change at *P*< 0.05 in comparison with the untreated DM group.

#### mRNA level of expression of pancreatic endocrine genetic markers

**Fig. 7:**
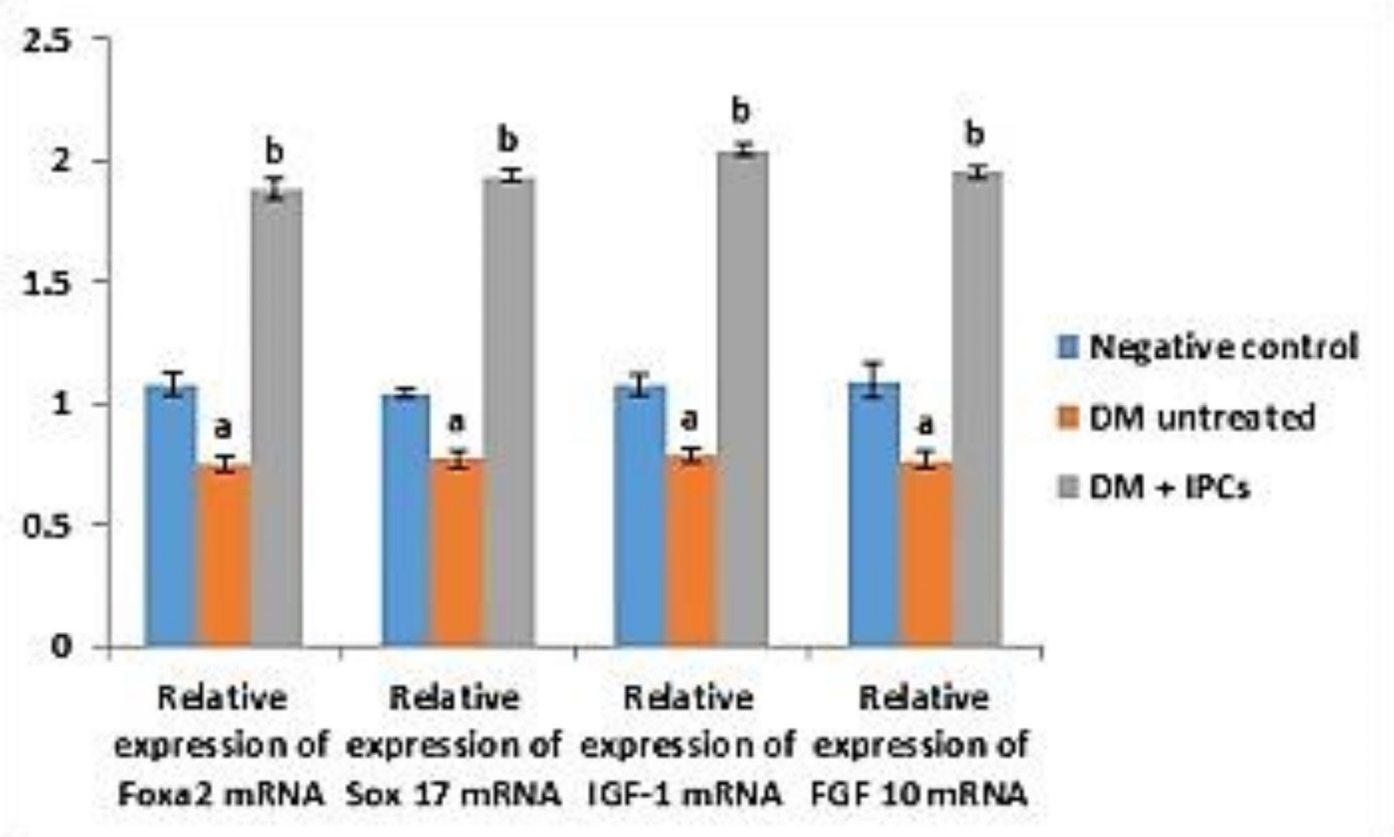
Influence of transfused IPCs on the expression level of pancreatic Foxa2, Sox 17, IGF-1 and FGF 10 genes in diabetic rats. Data are expressed as Means ± SD of 8 rats/group. ^a^Significant change at *P* < 0.05 in comparison with the negative control group. ^b^Significant change at *P* < 0.05 in comparison with the untreated DM group.

Pancreatic forkhead box protein A2 (*Foxa2*) and SRY-box transcription factor 17 (*Sox-17*), insulin-like growth factor I (*IGF-1*), fibroblast growth factor10 (*FGF 10*), and pancreatic glucagon (*GC*), levels of expression were estimated by qRT-PCR after in vivo injection of IPCs obtained from cells exposed to 3^rd^ protocol ingredients. Analytical data illustrated by Fig. 7, revealed significant up regulation (*P* < 0.05) of estimated genes expressed by group 3 of experimental animals (IPCs- treated diabetic rats) in relation to untreated diabetic group (group 2). The untreated diabetic rats (group 2) showed significant (*P* < 0.05) down-regulation in the expression level of pancreatic estimated genes in comparison with the control group.

#### Histological diversity of dissected pancreatic parenchyma

**Fig. 8:**
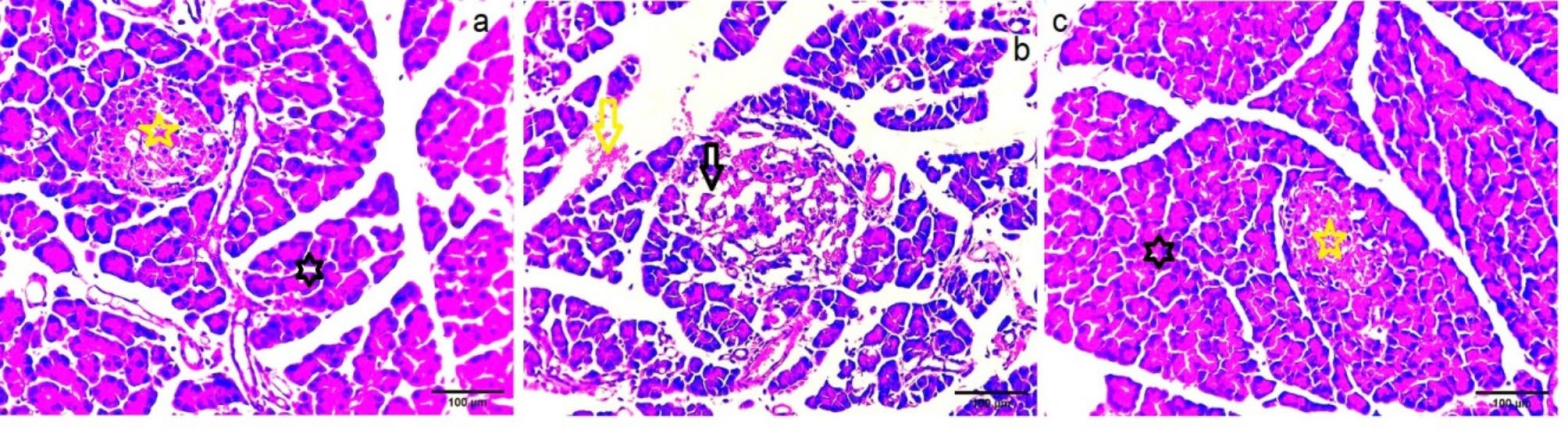
Histological section of pancreas tissue. (**a**) Negative control rat showed normal consistency of pancreatic tissue (black star) and regular islets of Langerhans (yellow star), (**b**) DM untreated rat showed atrophic irregular islets of Langerhans (black arrow) and hemorrhage occurred into expanded interlobular septa (yellow arrow) and (c) DM infused with IPCs rat revealed restored pancreatic parenchyma (black star) with regular appearance of islets of Langerhans (yellow star).

Histological examination of pancreatic tissue of normal control rat displayed normal pancreatic architecture of islets of Langerhans which organized of ribbed cells (Fig. 8a). It was found that pancreatic consistency of untreated diabetic rat was completely different with appearance of atrophic irregular acini with shrunken cells (Fig. 8b). While, pancreatic tissue examination of IPCs-treated diabetic rats revealed gradual restoration of normal islet cell configuration with normal tissue architecture (Fig. 8c).

## Discussion

ADSCs are considered to be the most eligible candidate for cell-based therapy. Their preference is due to accessible harvest for several times using minimal surgical interventions with low morbidity rate. The immunomodulatory effect of ADSCs is now evidenced giving them potent therapeutic role in the field of allogenic cell transfusion. The regenerative properties of MSCs are gained from their differentiation efficacy, and paracrine activity^29^. It was reported that MSCs transplantation could restore tissue damage through their interaction with the surrounding microenvironment promoting their secretion of exosomes containing biomolecules and growth elements which are substantial for angiogenesis, cell proliferation and differentiation ameliorating tissue repair process^30^.

Our results displayed that ADSCs fulfilled all MSC characteristics. According to the International Society for Cellular Therapy (ISCT), the isolated population of cells must be investigated for certain intrinsic features to be considered as mesenchymal stem cells^31^. We successfully harvested and cultured approximately homogenous ADSCs population emphasized morphologically, as the isolated cells exhibited typical fibroblast-like morphology, with adherence prospect to plastic culture flasks, and colony formation potential. Moreover, analysis of cell surface markers by flow cytometry revealed, positive expression of CD 73 (90.5%), CD 90 (96.5%) and CD 105 (92.2%) but they were negative for CD 14 (9.27%), CD 34 (2.12%) and CD 45 (8.95%)^32^. Finally, in vitro tri-lineage differentiation potential of the ADSCs was detected by lineage- specific stain observed under microscope at the end of induction protocols. Adipogenic differentiation was demonstrated as intracellular lipid droplets stained by Oil Red O stain. Calcium deposition and mineralized nodules was verified by Alizarin Red stain denoting osteogenic differentiation. While, chondrogenic differentiation appeared in the form of deposition of sulfated proteoglycans which was illustrated by Alcian blue staining. Proportionally homogeneous ADSCs population provided baseline step for further study protocols.

Effectiveness of stem cell differentiation is adjusted by both internal genetic programming and external inducers from the microenvironment. The process of cellular differentiation depends on activation of certain signaling pathways which is modulated *via* cell-to-cell and cell-matrix interactions. Several stages were involved in pancreatic differentiation starting from early progenitor cell commitment up to mature endocrine cell acquire^33^. Understanding of the developmental stages of pancreas during embryogenesis, is the key hallmark for establishment of differentiation strategies. The combination of target-specific growth factors and devoted small molecules is utilized to structure proper microenvironment suitable for induction of IPCs from stem cells of different origins^34^.

Three distinct protocols of differentiation were assessed in the current study. High glucose culture media was the baseline standard media for establishment of the assessed protocols. It was reported that high glucose concentration was considered as a robust initiator for pancreatic islet differentiation, as previous studies proved that it could alone induce IPCs formation^35^. Nicotinamide (NA) was used at different stages along with other growth factors during establishment of the selected protocols, as NA was commonly used as extrinsic induction factor for IPCs generation. Different studies have reported its critical role in differentiation of pancreatic progenitors into mature β-like cells^36,37^.

The first protocol of induction was based on pancreatic progenitor differentiation over 10 days using nicotinamide augmented by β-mercaptoethanol in high glucose culture, followed by final maturation to beta-like cells which was achieved by nicotinamide combined with exendin-4. Our data showed significant release of insulin in response to glucose challenge associated with insignificant (*P* < 0.05) up-regulation in the expression level of key pancreatic marker genes, *FOXa-2*,* SOX-17*,* PDX-1*,* NGN-3*, insulin and glucagon genes relative to undifferentiated ADSCs. Several studies elucidated the role of exendin-4 in addition of nicotinamide and β-mercaptoethanol in promoting MSCs differentiation of different sources towards IPCs^38^. The efficiency of the applied protocols varies widely depending on the source and type of stem cells selected for differentiation, and validating methods. Insulin release response of the differentiated cells is the most common in vitro parameter estimating differentiation. Our results were in concomitant with most of the previous studies however that insulin content was much lower than that of other selected protocols in the current study.

Basement membrane matrix could be useful for amelioration and stabilization of the propagated phenotype in different cell cultures including stem cells. In our second protocol, Geltrex was used as a substrate to support all of the differentiation stages throughout this protocol. Combination of activin A, retinoic acid (RA), basic fibroblast growth factor (bFGF), NA, and exendin-4 were used during the differentiation protocol steps. It was reported that, retinoic acid (RA) enhanced pancreas development and was widely used to induce pancreatic differentiation of ESC^39^. Activin A was considered as a chief regulator of organogenesis especially the pancreas. It was illustrated that its association with other inducer factors could augment its function promoting proliferation of induced insulin secreting cells and increased its hormonal content. Fibroblast growth factor (FGF) also plays a pivotal action in developing the pancreatic tissue. Previous studies highlighted the combined action of fibroblast growth factor and retinoic acid to direct efficient differentiation of PDX1 + foregut endoderm^40,41^. Our analytical studies revealed that the generated cells showed significant (*P* < 0.05) up-regulation in the expression level of *FOXa-2*,* SOX-17*,* PDX-1*, and glucagon genes and insignificant (*P* < 0.05) up-regulation in the expression level of *NGN-3* and insulin genes in comparison to the undifferentiated cells. The insulin release in response to glucose stimulation was significantly detected.

Generally, extracellular matrix (ECM) contains fibronectin, laminin, and collagens can profoundly influence stem cell fate choices^42^. Laminin was found to support mouse embryonic stem cells (ESCs) proliferation in vitro^43^ and soluble laminin could increase insulin secretion by 20% in murine islets in vitro^44^. In islet transplantation, treating islets with laminin prior to transplantation will help to maintain insulin production until new capillaries are formed in transplanted islets. Murine insulinoma cell line MINI6 cultured in laminin 411 could increase the insulin gene expression significantly^45^. In the current study, qRT-PCR and secretory functions of the generated IPCs using the ingredients of protocol (P3) and laminin as ECM, showed significant (*P* < 0.05) expression of pancreatic endocrine genes *versus* undifferentiated ADSCs, and higher insulin secretion in the laminin-coated plate group. Our results revealed that laminin gathered with insulin-transferrin-selenium, B27, N2, and nicotinamide, could efficiently induce the expression of early endoderm markers *FOXa-2*, and *SOX-17*, pancreatic differentiation marker, *PDX-1*, and eventually, *NGN-3*, insulin and glucagon, mature islet cell markers, were significantly upregulated. The proliferative action of N2 and B27 with maintenance of low apoptosis rate of stem cells was previously reported^46^. In addition, insulin-transferrin-selenite (ITS) participates in increasing nestin-positive cells, which could later propagate to insulin expressing cells^47,48^ The referred data illustrated that media containing these extrinsic factors along with the use of laminin as inducing extracellular matrix, could differentiate ADSCs into cells having a beta cell phenotype. According to the mentioned results, the differentiation potential of ADSCs to IPCs using laminin as ECM in coordination with other soluble factors, was the most eligible protocol of induction. Hence, further in vivo phase of the current study involved IPCs produced by laminin-dependent protocol.

Teshima et al.^49^ registered that, spheroid-like small clusters consisting of canine ADSCs and human recombinant peptide *µ*-pieces developed under a three-dimensional (3D) culture system were successfully differentiated into IPCs. The generated IPCs under 3D culture condition showed significant upregulation of the *PDX-1*, *NGN-3*, and *GLUT2* mRNAs expression and increased insulin secretion in response to glucose stimulation. Furthermore, in Hosseini et al.^50^ study, human ADSCs were used to differentiate into IPCs on a silk/polyethersulfone (PES) scaffold. They recorded that, after exposing to the differentiation media, 2D and 3D (silk/PES) cultured cells were gradually aggregated and formed spherical shaped clusters. The viability of cells was comparable in both 3D and 2D culture and the results of *PDX-1*, *Glut-2*, *NGN-3*, glucagon, and insulin genes expression showed the differentiation efficiency was higher in 3D culture. Ojaghi et al.^51^ found that, the expression level of *PDX-1*, *Glut-2*, *NGN-3*, glucagon, and insulin genes in the poly (L-lactic acid/polyvinyl alcohol (PLLA/PVA) nanofibers as a 3D scaffold-seeded differentiated cells was at the highest level compared with the 2D culture. Also, the results of Piran et al.^52^ study were shown that insulin and *PDX-1* genes and proteins expression significantly increased in ADSCs treated by lentiviruses containing miR-375, compared to the control group. The superior impact of 3D culture on the differentiation efficiency of stem cells into IPCs stems from the efficacy of scaffold in boosting the differentiation competence of stem cells due to providing a supportive matrix to mimic 3D in vivo microenvironment^53^.

The effectiveness of stem cell-derived IPC therapy on diabetes was speculated by animal studies. STZ-induced diabetic rats were used as standard model to evaluate the ability of these cells in management of diabetes. The homing efficacy of the systemically infused therapeutic cells was extensively studied to ameliorate the cell therapy potential in regenerative medicine. It was reported that the paracrine and juxtacrine effect of the systemically infused cells enhance the regenerative properties after reaching target organ or tissue^54^. According to our results, successful homing of the PKH-26-labelled IPCs was detected in the pancreas of treated animal group. These results were reconciled with the previous published studies demonstrated that differentiated mesenchymal stem cells could home to the target organ efficiently^55^. In vivo release of insulin and C-peptide were used as indicators of pancreatic cell functionality. The metabolic parameters estimated in the studied groups revealed significant (*P* < 0.05) elevation of serum INS (10.1 ± 2.2 mU/ l) and c-peptide (728.1 ± 194.9 pg /ml), which improve the condition of hyperglycemia (310.9 ± 53.6 mg/ dl) detected in the untreated STZ-induced diabetic rats, as the fasting blood glucose reached to be 112.4 ± 21.3 mg/ dl, but not reaching the normoglycemic state of the negative control group (96.5 ± 9.45 mg/ dl). The serum content of insulin and c-peptide of the untreated group were significantly declined to be 4.1 ± 0.97 mU/ l, and 370.6 ± 71.8 pg /ml, respectively, by contrast with that of the negative control, 13.5 ± 2.86 mg/ dl, and 897.5 ± 67.1 pg /ml, respectively. Visfatin is a newly discovered adipokine hormone with a direct relationship between plasma visfatin level and type 2 diabetes mellitus. It causes hypoglycemia by suppressing glucose release from liver cells and promoting glucose utilization in adipocytes and myocytes. Visfatin is upregulated by hypoxia, inflammation and hyperglycemia and downregulated by insulin, somatostatin and statins. According to our results, visfatin was significantly (*P* < 0.05) upregulated in the diabetic rats receiving no IPCs (244.9 ± 24.2 ng/ml) contrary to the negative control group (156.3 ± 10.6 ng/ml), while, IPCs-treated diabetic rats presented significantly declined visfatin level (165.4 ± 12.7 ng/ml). Glucagon hormone is released from α- cells and works in concomitant with insulin to ensure glucose homeostasis. Subsequently the estimated glucagon level in the pancreatic tissue of the untreated diabetic rats showed significant increase (320.8 ± 22.5 pg /g tissue) relative to the negative control (161.3 ± 21.6 pg /g tissue), whereas that level was relatively declined in the pancreatic tissue of IPCs- infused rats (202.5 ± 28.4 pg /g tissue). These findings coincided with the previous research that revealed that the infusion of laminin 411-induced IPCs rapidly and significantly downregulated fasting blood glucose levels, significantly reduced the HbA1c concentration, and markedly ameliorated the symptomatic manifestations of diabetic rats^19^. Furthermore, IPCs infusion in diabetic rats provoked significant (*P* < 0.05) up-regulation in the level of expression of pancreatic related genes, *Foxa2*,* Sox 17*,* IGF-1* and *FGF 10* when compared with the untreated DM rats. Moreover, histological examination of dissected pancreatic tissue revealed the regenerative effect of infused IPCs in the form of gradual restoration of normal islet cell configuration with normal tissue architecture. It was previously elucidated that in vitro generated IPCs could reverse the hyperglycemia in animal models^56^.

## Summary and conclusion

To sum up, diabetes mellitus (DM) is a comprehensive metabolic disease with significant prevalence rate across the world. Recently, stem cells of various origins are represented as the savior management of diabetes mellitus. To generate clinically efficient IPCs, proper stem cell type, defined induction protocols, appropriate recipe of particular extrinsic factors and small molecules, and extracellular matrix composition, should be considered carefully^57^.

Many research interestingly highlighted that, ADSCs are distinctively applicable rather than other MSC-sources, as these cells can be obtained in abundance with minimally invasive procedures. In addition, ADSCs pose remarkable immunomodulatory effect, with threefold increase in immunosuppressive activity, and high differentiation potential in relation to their counterparts^58,59^.

Islet –like cell differentiation from MSC builds up on the basis of two major steps. Firstly, the cells are differentiated into pancreatic progenitors followed by beta cell maturation. Pancreatic progenitor differentiation was promoted mostly by using nicotinamide with or without growth factors or peptides in high glucose culture. In addition, chemicals like acivin A, insulin transferrin selenium and sodium butyrate also potentiated the endocrine differentiation of MSC. The key genetic markers analyzed during the pancreatic progenitor stage are PDX1, NKX6.1, and Ngn3. The final maturation to beta-like cells was obtained by nicotinamide combined with exendin-4 or glucagon-like peptide-1 (GLP-1), and the vital genes analyzed included ISL1, insulin, and c-peptide^37^.

Eventually, IPCs generation procedure is a multi-step induction protocol with quite variable induction period depending on the type of cells used, as it may require several days up to several months^60^. Furthermore, supplement and withdrawal of inducing soluble factors in a stage-wise pattern must be carefully speculated to boost beta-cell proliferation and differentiation and increment insulin content of acquired IPCs.

In the current study, the isolated fibroblast-like cell population was successfully fulfilling MSCs standard criteria. Next, ADSCs were subjected to three distinct protocols for induction towards IPCs. The results of the applied selected protocols were quite different than that of reference literature. The variation between results could be due to the use of diverse cell source and different cell number to start with. Nevertheless, the synergistic effect of using laminin–coated plates in concomitant with extrinsic factors promoting proliferation and differentiation of ADSCs, revealed interestingly promising consequences. The in vitro outcomes were documented by significant insulin release in response to glucose challenge, and upregulation of expression levels of pancreatic gene markers. Furthermore, the in vivo animal studies revealed missionary evidence for the in vivo effect of transplanted IPCs in the form of restoration of hyperglycemic state and metabolic changes of STZ-induced diabetic rats, with regain of pancreatic tissue organization detected by histological examination. Notably, establishment of suitable microenvironments with appropriate extracellular matrices, metabolites, and small molecules or growth factors are required for efficient cell therapy. However, several challenges are still facing scientists to acquire clinically significant amounts of IPCs. It is not confirmed whether the differentiated cells (IPCs) that could ameliorate hyperglycemia in animal models can correct hyperglycemia in human subjects.

### Limitations of the study

Cell viability assay should have performed to confirm the final cell number after applying different differentiation protocols. Moreover, we didn’t demonstrate the hyperglycemic reversal in time-dependent manner in animals. So, further investigations including immunostaining of IPCs with insulin antibody may be done in the future.

### Future recommendations

Different strategies, such as improvements of the conditioned media by adding inducible factors and using bio-compatible 3D scaffolds can be endeavored to obtain long-term functional ability of pancreatic β-like cells.

## Data Availability

The datasets used and/or analyzed during the current study are available from the corresponding author on reasonable request.
